# Research on the evolution of spatial network structure of tourism eco-efficiency and its influencing factors in China’s provinces based on carbon emission accounting

**DOI:** 10.1371/journal.pone.0272667

**Published:** 2022-09-14

**Authors:** Chao Wang, Lele Xu, Menglan Huang, Xiaofeng Su, Riwen Lai, Anxin Xu

**Affiliations:** 1 College of Forestry, Fujian Agriculture and Forestry University, Fuzhou, Fujian, China; 2 Anxi College of Tea Science, Fujian Agriculture and Forestry University, Anxi, Fujian, China; 3 College of Economics and Management, Fujian Agriculture and Forestry University, Fuzhou, Fujian, China; 4 College of Administrative Management, Fujian Business University, Fuzhou, Fujian, China; Northeastern University (Shenyang China), CHINA

## Abstract

In the context of global warming, although the coordinated development of tourism has led to regional economic growth, the high energy consumption-driven effects of such development have also led to environmental degradation. This research combines the undesired output of the Super-SBM model and social network analysis methods to determine the eco-efficiency of provincial tourism in China from 2010–2019 and analyzes its spatial correlation characteristics as well as its influencing factors. The aim of the project is to improve China’s regional tourism eco-efficiency and promote cross-regional tourism correlation. The results show that (1) the mean value of provincial tourism eco-efficiency in China is maintained at 0.405~0.612, with an overall fluctuating upward trend. The tourism eco-efficiency of eastern China is higher than that of central, western and northeastern China, but the latter three regions have not formed a stable spatial distribution pattern. (2) The spatial network of provincial tourism eco-efficiency in China is multithreaded, dense and diversified. Throughout the network, affiliations are becoming closer, and network structure robustness is gradually improving, although the “hierarchical” spatial network structure remains. In individual networks, Jiangsu, Guangdong and Shandong provinces in eastern China have higher centrality degrees, closeness centrality and betweenness centrality than other provinces, which means they are dominant in the network. Hainan Province, also located in eastern China, has not yet built a "bridge" for tourism factor circulation. In the core-periphery model, the core-periphery areas of China’s provincial tourism eco-efficiency are distributed in clusters, and the number of "core members" has increased. (3) The economic development level, information technology development level, and tourism technology level collectively drive the development and evolution of China’s provincial tourism eco-efficiency spatial network.

## 1 Introduction

Tourism is recognized as the world’s first-ranked industry [1] and has become the leading industry in many countries or regions due to its rapid economic growth rate and strong multiplier effect [2], which can effectively drive regional economic development and help regions escape poverty [3, 4]. The World Travel & Tourism Council (WTTC) has pointed out that the total value of tourism in 2019 accounted for approximately 10.4% of the global GDP [5]. Nevertheless, tourism, as a highly energy-intensive industry, releases large amounts of greenhouse gases from large-scale tourist flows and tourism activities [6–8], further contributing to global climate change [1, 9]. The World Tourism Organization (UNWTO) has stated that carbon emissions from tourism account for 5%–14% of total carbon emissions from human activities [10]. Carbon emissions have become an important measurement of the environmental impact of the tourism industry [6, 8, 11, 12]. Consequently, to address the contradictory relationship between environmental protection and economic development in the field of tourism [13], the concept of the eco-efficiency of sustainable development was introduced into the field of tourism research [14]. As one of the important indices to measure the coordination degree of the regional man-land system and the development level of sustainable tourism, tourism eco-efficiency represents the two-dimensional relationship of "economy-environment"; it is of great theoretical value and practical significance to solve the dual contradiction and promote tourism to advance high-quality green development.

The concept of eco-efficiency was first proposed in 1990 and refers to the minimization of resource input and environmental damage and the maximization of socioeconomic benefits [15]. As a result, Tourism eco-efficiency is derived from it [16]. The current research related to tourism eco-efficiency focuses on four aspects: core connotation and theoretical framework [16–18], measurement evaluation and empirical analysis [14, 19], spatial phenomenon characterization [20, 21], and exploration of influencing factors and driving mechanisms [22–24]. In terms of its core connotation, most scholars consider tourism eco-efficiency as a tourism environmental performance to increase tourism resource productivity [16]. It is a tool to characterize the "maximization of tourism economy and minimization of environmental stress" and represents the vision of sustainable development of tourism sites [25]. For this reason, measuring and evaluating tourism eco-efficiency has become one of the research priorities. Scholars have focused on tourism economic development (total tourism revenue and total number of tourists) [14], tourism environmental impact (tourism waste, ecological footprint) [26], tourism labor force [27], tourism energy consumption [28], and tourism fixed assets (hotels, travel agencies, etc.) [14], using single indicator approach (described by the ratio of indicators representing the value of products and services to indicators representing the environmental load) [29], life cycle assessment [30], and data envelopment analysis (DEA) [31, 32] to measure tourism eco-efficiency. It should be noted that the DEA method considers the combined efficiency of multiple industry inputs and outputs. It is independent of the input–output index scale, which suggests an improvement direction for inefficient decision-making units (DMU). Therefore, the DEA model is widely used in the field of tourism [14]. However, the traditional DEA model ignores factor slack and undesired output indicators and directly causes bias in the efficiency measures, which affects the evaluation and judgment of the decision unit [33]. The super-efficiency slacks-based measure (Super-SBM) model overcomes the shortcomings of the traditional model very well [34]. Thus, we incorporate tourism carbon emissions into the Super-SBM model to calculate tourism eco-efficiency.

To explore the tourism eco-efficiency spatial distribution pattern, some scholars have used "attribute data" (defined as data specific to the "actor" itself) from different research scales, such as economic belts [35], provinces [22], urban agglomerations [23], and scenic spots [14], and have employed spatial correlation analysis [36], Getis-Ord Gi* [37], standard deviation ellipses [38] and geodetectors [20] to explore spatial distribution patterns, central dispersion and directional trends, spatial stratification heterogeneity and other spatial characteristics. However, spatial measurement based on "attribute data" can only examine the types and characteristics of regional tourism eco-efficiency spatial aggregation; it is difficult to explore changes in the spatial network structure of tourism eco-efficiency and the degree of tourism eco-efficiency relationships and connections among regions, and it is even more difficult to clarify the different roles played by each region in the network space from the perspective of "relational data" (defined as data related to associations) [39]. Unevenly distributed tourism resources lead to provinces being both tourism sources and destinations. The material and information flows generated by interprovincial tourism flows form a massive network of spatial relationships. Mining the spatially linked network characteristics of tourism eco-efficiency in China’s provinces is beneficial for promoting interprovincial tourism spatial integration strategies [40–42]. Among the existing studies, only a few explore the spatial network of tourism eco-efficiency based on "relational data", such as the Yangtze River Delta city [21]. However, it is difficult to grasp the spatially integrated development characteristics of China’s provincial tourism eco-efficiency as a whole by setting a municipal area as the research scale. Accordingly, in order to explain the phenomenon of spatial differentiation and enhance tourism eco-efficiency, scholars have devoted themselves to seeking its influencing factors. At present, scholars mostly explore the influence of economic development level [43], tourism industry structure [14], government environmental regulation and digital technology on tourism eco-efficiency based on linear regression [44, 45]. However, the influencing factors of tourism eco-efficiency networks based on relational data are less explored. Social network analysis (SNA) uses graph-theoretic tools and algebraic linear modeling techniques to describe relationship patterns and to explore the impact of relationship patterns on the members of the structure or the whole [46]. In the 1990s, SNA was introduced into tourism research [39] but was applied mainly to the fields of disaster management studies [35], stakeholder networks [47], and tourism flow networks [48]. This study uses SNA to study the spatial network of tourism eco-efficiency in China’s provinces.

In view of the above discussion, this research investigates the spatial network and influencing factors of the tourism eco-efficiency structure in China’s provinces. Thirty provinces (excluding Tibet, Macao, Hong Kong and Taiwan) of China (cities and districts) from 2010–2019 are used as target areas for the study, and the undesired output Super-SBM model is applied to measure the tourism eco-efficiency values in the provinces during the research period in terms of labor input, fixed asset input, tourism energy input, economic benefits, social benefits, and tourism carbon emissions. The "relational data" obtained from the gravitational model are used to construct a spatial correlation matrix of tourism eco-efficiency in China’s provinces and reveal its spatial structural characteristics through SNA. Furthermore, quadratic assignment procedure (QAP) correlation analysis and regression analysis are used to clarify the key factors driving the optimal reorganization of the tourism eco-efficiency network structure. This study provides a basis for a spatial integration strategy for the eco-efficiency of China’s provincial tourism and a scientific reference for the green development of China’s provincial tourism.

**Fig 1:**
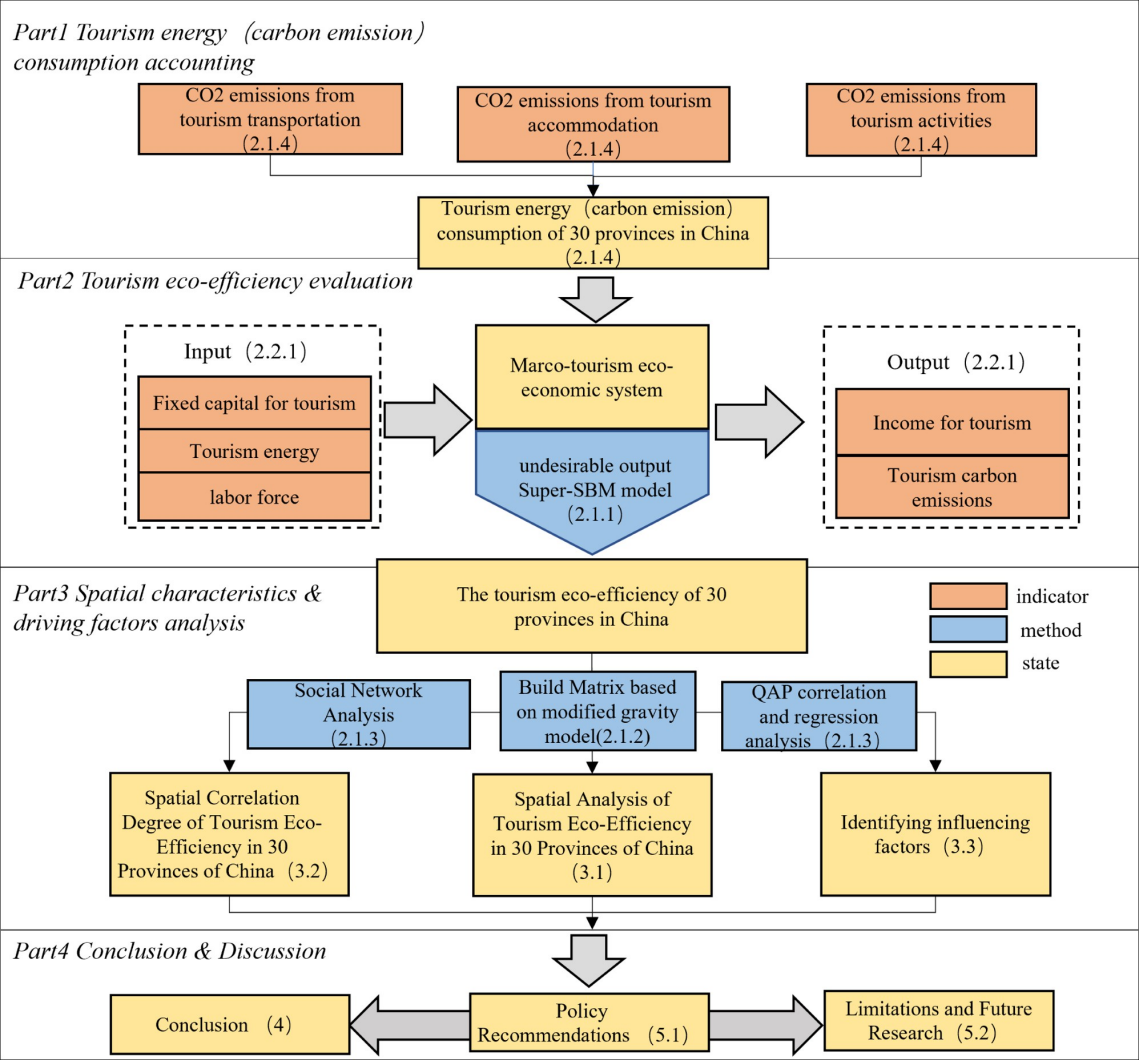
The technical route applied for China’s provincial tourism eco-efficiency research.

This article is structured as follows. Section 1 introduces the research. Section 2 presents the research materials and methods. Section 3 describes the results and analysis, including the spatial distribution pattern of tourism eco-efficiency, the analysis of the tourism eco-efficiency spatial network and the analysis of the factors affecting the spatial network of tourism eco-efficiency. Section 4 offers the conclusions, and Section 5 presents the discussion (Fig 1).

## 2 Materials and methods

### 2.1 Methods

#### 2.1.1 Undesired output super-SBM model

Since the traditional DEA model cannot address bias and impact due to radial and angular selection, The Super-SBM model is a powerful solution to this problem [34]. When the efficiency value of multiple decision units is 1, traditional DEA models cannot compare and rank these relatively efficient decision units; thus, further optimization obtains the Super-SBM model. The specific model is as follows:

$$\left\{\begin{array}{c} min\rho =\left(1-\frac{1}{\text{m}}\overset{\text{m}}{\underset{\text{i}=1}{\sum }}\frac{{\text{s}}_{\text{i}}^{-}}{x_{ik}}\right)/\left[1+\frac{1}{{\text{q}}_{1}+{\text{q}}_{2}}\left(\overset{{\text{q}}_{1}}{\underset{\text{r}=1}{\sum }}\frac{{\text{s}}_{\text{r}}^{+}}{y_{\text{rk}}}+\overset{{\text{q}}_{2}}{\underset{\text{r}=1}{\sum }}\frac{{\text{s}}_{\text{t}}^{\text{b}-}}{y_{tk}}\right)\right]\\ \text{s}.\text{t}.\\ x_{\text{k}}=X\lambda +{\text{s}}^{-},{\text{y}}_{\text{k}}=Y\lambda -{\text{s}}^{+},b_{\text{k}}=b\lambda +{\text{s}}^{\text{b}-}\\ \lambda ⩾0,{\text{s}}_{i}^{-}⩾0,{\text{s}}_{r}^{+}⩾0,{\text{s}}_{t}^{\text{b}-}⩾0 \end{array}\right.$$ (1)

Where *ρ* is the efficiency value, and m, q_1_ and q_2_ are the number of indicators for input, desired output and undesired output, respectively. *x*_*k*_, *y*_*k*_ and *b*_*k*_ denote input variables, desired output variables and undesired output variables of the evaluated decision unit, respectively, and *x*_*ik*_, *y*_*rk*_ and *y*_*tk*_ are elements of the input and output vectors. *X*, *Y* and *b* are input–output matrices; $S_{i}^{-};S_{r}^{+}$ and $S_{t}^{b-}$denote the input, desired output and undesired output slack variables, respectively, and λ is the column vector.

#### 2.1.2 Modified gravity model

Interregional economic relations have a law similar to that of gravity; that is, the strength of interregional relations is directly proportional to the "quality" of the region and inversely proportional to the "distance" between regions [40]. This research explores the relation of tourism eco-efficiency among 30 provinces in China, so the "quality" of the region is regional tourism eco-efficiency. The research uses a modified gravity model to develop a tourism eco-efficiency correlation matrix among China’s provinces. The specific model is as follows:

$$F_{\text{ij}}={\text{K}}_{\text{ij}}\frac{{\text{E}}_{\text{i}}\cdot {\text{E}}_{\text{j}}}{D_{\text{ij}}{}^{2}},{\text{K}}_{\text{ij}}=\frac{E_{\text{i}}}{E_{\text{i}}+E_{\text{j}}},D_{\text{ij}}{}^{2}={\left(\frac{{\text{d}}_{\text{ij}}}{{\text{g}}_{\text{i}}-{\text{g}}_{\text{j}}}\right)}^{2}$$ (2)

where *F*_*ij*_ is the intensity of the tourism eco-efficiency linkage of each province in China, and K_ij_ is the gravitational coefficient. E_i_ and E_j_ indicate the tourism eco-efficiency of province i and province j, respectively. *D*_*ij*_ represents the "economic distance" between province i and province j. d_ij_ is the spherical distance between province i and province j. g_i_ and g_j_ denote the GDP of province i and province j, respectively. The spatial correlation matrix of China’s provincial tourism eco-efficiency is constructed from the gravity model, and the mean values of the data in each row of the matrix are binarized as threshold values and then converted into a binary matrix. If *F* is greater than the threshold, then the value is 1, indicating that there is a spatial correlation between the row and the tourism eco-efficiency of the column province; conversely, the value of 0 indicates that there is no spatial correlation between the row and the tourism eco-efficiency of the column province.

#### 2.1.3 Social network analysis

SNA is a sociological approach from a structural perspective, the core of which is to study structural issues from a "relational" perspective. In this research, network density, network connectedness, network hierarchy and network efficiency are selected to analyze the overall network structure of tourism eco-efficiency characteristics in China’s provinces. In the individual network, degree centrality, closeness centrality and betweenness centrality are selected to quantify the centrality of each province as a reflection of the province’s rights in the tourism eco-efficiency network. The "core-periphery" model reflects the position of a province in the network structure. Furthermore, QAP correlation analysis and regression analysis are used to identify the key factors affecting the spatial correlation strength of tourism eco-efficiency in China’s provinces. The analysis is based on the permutation of matrix data, and the estimation process is roughly divided into 3 steps. First, a conventional multiple regression analysis (correlation analysis) is performed on the corresponding long vector elements of the matrix to obtain the actual parameter estimates. Second, the rows and columns of the matrix are randomly permuted and re-estimated, keeping all estimated coefficients. Finally, the permutation step is repeated enough times to calculate the proportion of random permutations that are greater than or equal to the actual parameter estimates among the total number of random permutations. Thus, the standard error of the statistic is estimated, and the significance test is completed [38, 49]. The theoretical formula of this study is as follows:

$$G=f\left(EDL,TIS,TRE,IDL,TTL\right)$$ (3)

where *G* is the tourism eco-efficiency spatial network matrix, *EDL* is the matrix of the economic development level, *TIS* is the tourism industry structure, *TRE* is the tourism resource endowment, *IDL* is the information development level, and *TTL* is the tourism technology level.

#### 2.1.4 "Bottom-up" model of tourism carbon emissions and tourism energy consumption

At present, no systematic approach has been developed globally for estimating energy consumption and CO_2_ emissions from tourism [7]. In existing studies, "top-down" [50] or "bottom-up" [14] approaches are often used. The "top-down" approach, which directly estimates the share of tourism energy consumption (tourism carbon emissions) in a system, requires energy consumption statistics or carbon dioxide emission monitoring data at the national/regional level. However, China has not yet established a national and regional statistical monitoring system for greenhouse gas emissions, so they are difficult to estimate in this way. The "bottom-up" approach is based on estimating energy consumption (carbon emissions) in each sector of the tourism industry and then summing them. In this research, we use the "bottom-up" method to estimate the tourism energy consumption and tourism carbon emission intensity of 30 provinces (cities and districts) in China based on the estimation method of tourism energy consumption (tourism carbon emissions) [50–52]. It should be noted that tourism energy consumption (tourism carbon emissions) comes from numerous sectors (food, accommodation, travel, activities, shopping and entertainment), but its transportation, accommodation and principal activities constitute the leading sources of energy consumption, so this study will account for the energy consumption and carbon emissions of the above three modules and sum them [16]. The specific model is as follows:

$$\begin{array}{c} {\text{C}}_{\text{transportation}}=\overset{\text{n}}{\underset{\text{i}=1}{\sum }}\left({\text{Q}}_{\text{i}}\times {\text{w}}_{\text{i}}\times \alpha_{\text{i}}\right)\\ {\text{C}}_{\text{accommodation}}=q\times s\times T\times \beta \\ {\text{C}}_{\text{activities}}=\overset{\text{n}}{\underset{\text{k}=1}{\sum }}\left({\text{P}}_{\text{k}}\times \gamma_{\text{k}}\right)\\ {\text{C}}_{\text{energy}\;\text{consumption}}={\text{C}}_{\text{transportation}}+{\text{C}}_{\text{accommodation}}+{\text{C}}_{\text{activities}} \end{array}$$ (4)

where C_transportation_ is tourism traffic energy consumption (carbon emissions); Q_i_ is the passenger turnover of railroad, road and water transport; and w_i_ indicates the proportion of tourists among passengers. Referring to the research results [9, 51, 53], the proportions of tourists among railroad, road and water transport are taken as 31.6%, 13.8% and 10.6%, respectively. α_i_ is the energy consumption coefficient (carbon emission factor) of railroad, road and water transportation, taking the values of 1, 1.8 and 1.48 MJ/(person-km), respectively. C_accommodation_ is tourism accommodation-related energy consumption (carbon emissions), in which q is the number of beds, s is the room occupancy rate, T is the number of days in a year (365 d is selected for this research), and β indicates the accommodation energy consumption coefficient (carbon emission coefficient); the tourism accommodation energy consumption coefficient is 155 MJ/(person-night), and the carbon emission factor is 245.8 g/(person-night). C_activity_ is tourism activity energy consumption (carbon emission), P_k_ represents various types of tourism activity person-times, and γ_k_ represents the activity energy consumption coefficient (carbon emission coefficient). Referring to the research results [51, 53] the energy consumption coefficients of sightseeing, leisure and vacation activities, visiting family and friends, business meetings and other types of tourism activities are 8.5, 26.5, 12, 16 and 3.5 MJ/person, respectively, and the carbon emission factors are classified as 417, 1670, 591, 786 and 172 g/person, respectively.

### 2.2 Indicators and data

#### 2.2.1 Indicator system

The core concept of eco-efficiency is the "minimum environmental impact, to produce the maximum economic output". The framework of the undesired output SBM model, in addition to concern for economic output, also includes undesired output indicators, such as environmental pollution. In terms of input indicators, current studies revolve mainly around the basic factors of capital, labor, and land. To this foundation, some scholars have added energy input and water consumption indicators. This study draws on the existing research results [14, 53] to consider the size of the tourism workforce as a labor input. It should be noted that, in view of the change in the caliber of statistics on the tourism workforce and the characteristics of the tourism industry, the population employed in the tertiary sector is treated as the tourism workforce. The number of travel agencies, the number of scenic spots classified as 3A level and above, and the number of 3-star and above accommodations are defined as capital investment. In addition, drawing on the research results [50, 51] tourism energy consumption is used in this study as an input indicator. The "bottom-up" energy accounting method is used to account for tourism energy consumption in tourism transportation, accommodation and activities. In the desired output, the study chooses total tourism revenue and total tourism visitors as the consensual output. In the case of undesired outputs, tourism carbon emissions are a core indicator of the environmental impact of tourism, accounted for using the "bottom-up" approach (Table 1).

**Table 1:** Evaluation index system of provincial tourism ecological efficiency in China.

Type	Primary Indicators	Secondary indicators	Specific target	unit
Input Indicators	Resource input	Workforce input	Number of tertiary sector workforce	Ten thousand
		Fixed assets input	Number of star-rated hotels	individual
			Number of travel agencies	individual
			Number of weighted scenic spots	individual
	Energy consumption	Tourism energy input	Tourism traffic energy consumption	PJ
			Tourism accommodation energy consumption	PJ
			Tourism activity energy consumption	PJ
Output Indicators	Desired output	Economic benefits	Total Tourism Revenue	Billion yuan
		Social benefits	Total number of visitors received	Billion people
	Undesired Output	Tourism Carbon Emissions	Tourism Transportation Carbon Emissions	Mt
			Tourism Accommodation Carbon Emissions	Mt
			Tourism Activities Carbon Emissions	Mt

#### 2.2.2 Data source

The tourism input and output panel data of China’s 30 provinces and regions (excluding Hong Kong, Macau, Taiwan and Tibet) from 2010 to 2019 are selected as the dataset for this research. Tourism workforce is from the China Statistical Yearbook. Total tourism revenue, the total number of tourists, the number of 3-star and above accommodations, the number of travel agencies and the number of 3A and above scenic spots are drawn mainly from the China Tourism Statistical Yearbook and the statistical yearbooks and statistical bulletins of each province (city and district). Both tourism energy consumption and tourism carbon emissions are calculated by the 2.1.4 "bottom-up" model of tourism carbon emissions and tourism energy consumption. To eliminate the effect of price fluctuations, all the above value indicators are deflated based on the Consumer Price Index (CPI) of the previous year, using 2010 as the base period. Some of the absent data are supplemented by linear interpolation for completeness. Referring to the existing research [54], the capital cities of the two provinces are taken as the centroids of the provinces, and the distance between the centroids is the distance between the two provinces. The center-of-mass distance is calculated by the "point distance" of ArcGIS 10.8 Proximity.

## 3 Results

### 3.1 Evolution of spatial differences in tourism eco-efficiency in China’s provinces

In accordance with the National Bureau of Statistics of the People’s Republic of China, the research divided China into east, central, west, and northeast (Table 2).

**Table 2:** East, West, Central and Northeast China divisions.

Region	Provinces
east	Beijing, Tianjin, Hebei, Shanghai, Jiangsu, Zhejiang, Fujian, Shandong, Guangdong, Hainan
central	Shanxi, Anhui, Jiangxi, Henan, Hubei, Hunan
west	Inne rmongolia, Guangxi, Chongqing, Sichuan, Guizhou, Yunnan, Shaanxi, Gansu, Qinghai, Ningxia, Xinjiang
Northeast	Liaoning, Jilin Heilongjiang

**Fig 2:**
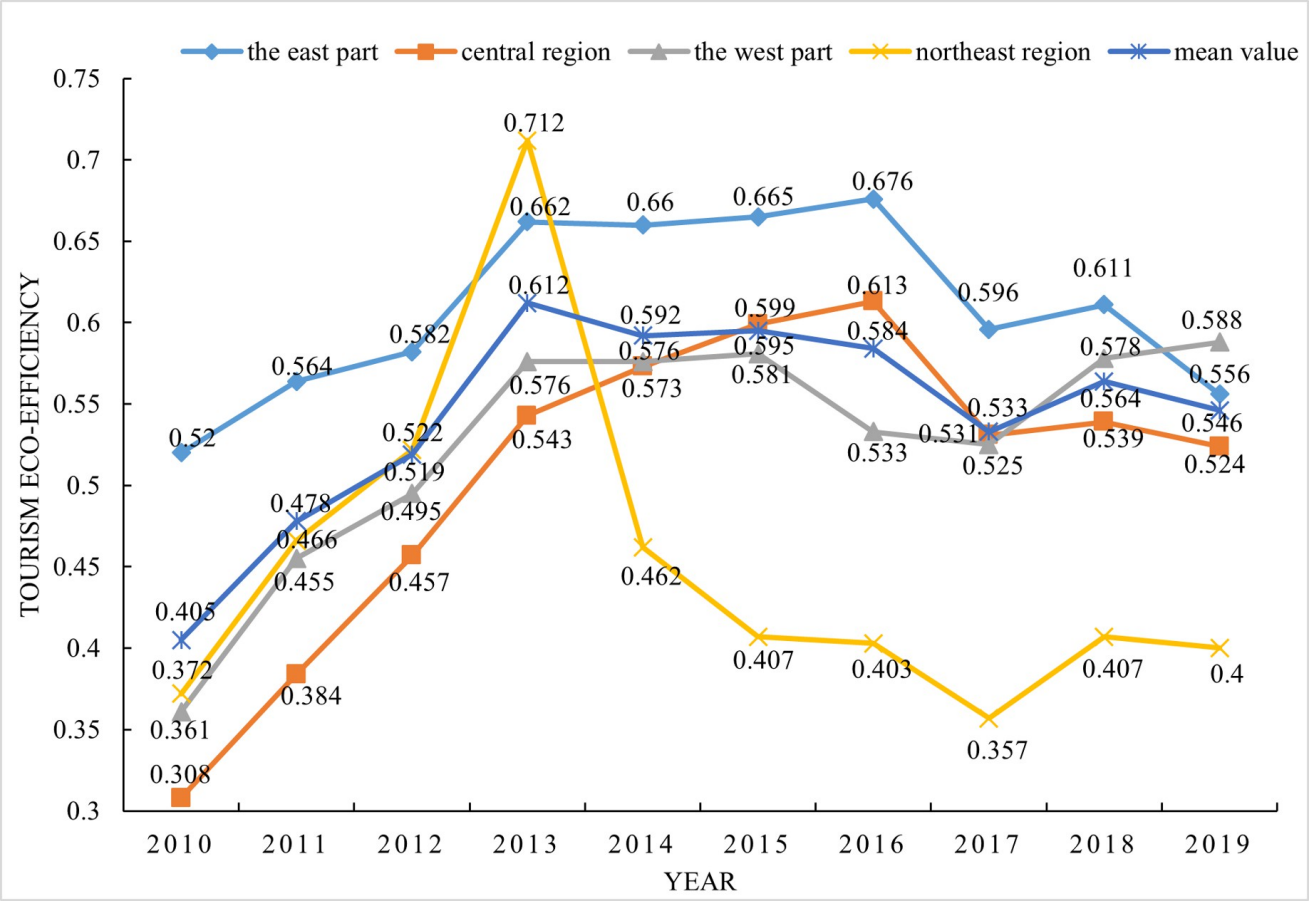
Eco-efficiency value of regional tourism in China from 2010–2019.

As shown in Fig 2, from 2010 to 2019, the mean value of tourism eco-efficiency in China was 0.543, and the mean value of efficiency was maintained at 0.405~0.612 for each year. In terms of temporal trends, tourism eco-efficiency showed an overall fluctuating upward trend, rising from 0.405 in 2010 to 0.546 in 2019, an increase of 34.8%. The fluctuating trend of tourism eco-efficiency in China’s provinces during the study period can be roughly divided into three phases: an increasing phase from 2010–2013, a decreasing phase from 2014–2016, and a fluctuating decreasing phase from 2017–2019. In terms of spatial distribution, the east has abundant tourism resources and mature green innovation technologies, making it the leader in tourism eco-efficiency among the other regions. Differentiated environmental regulation policies have also resulted in an unstable spatial distribution pattern between the northeastern, western and central regions. The east showed stable development, with tourism eco-efficiency values at 0.52 and above. The central tourism eco-efficiency value showed rapid growth, followed by a slow decline trend. In 2016, the watershed year, the tourism eco-efficiency value is 0.613. Correspondingly, as the economic development of the western region in China took place, the economic structure became increasingly rational, and dividends emerged. Tourism eco-efficiency in the western region showed steady growth during the research period, with values ranging from 0.361–0.588. The northeast, in contrast, experienced rapid growth from 2010–2013, while a major slide occurred from 2014–2019. This decline may be related to the industrial structure of the northeast, which houses mainly energy power plants and has experienced serious population loss, resulting in few inputs of tourism resource elements and a rough tourism development model.

**Fig 3:**
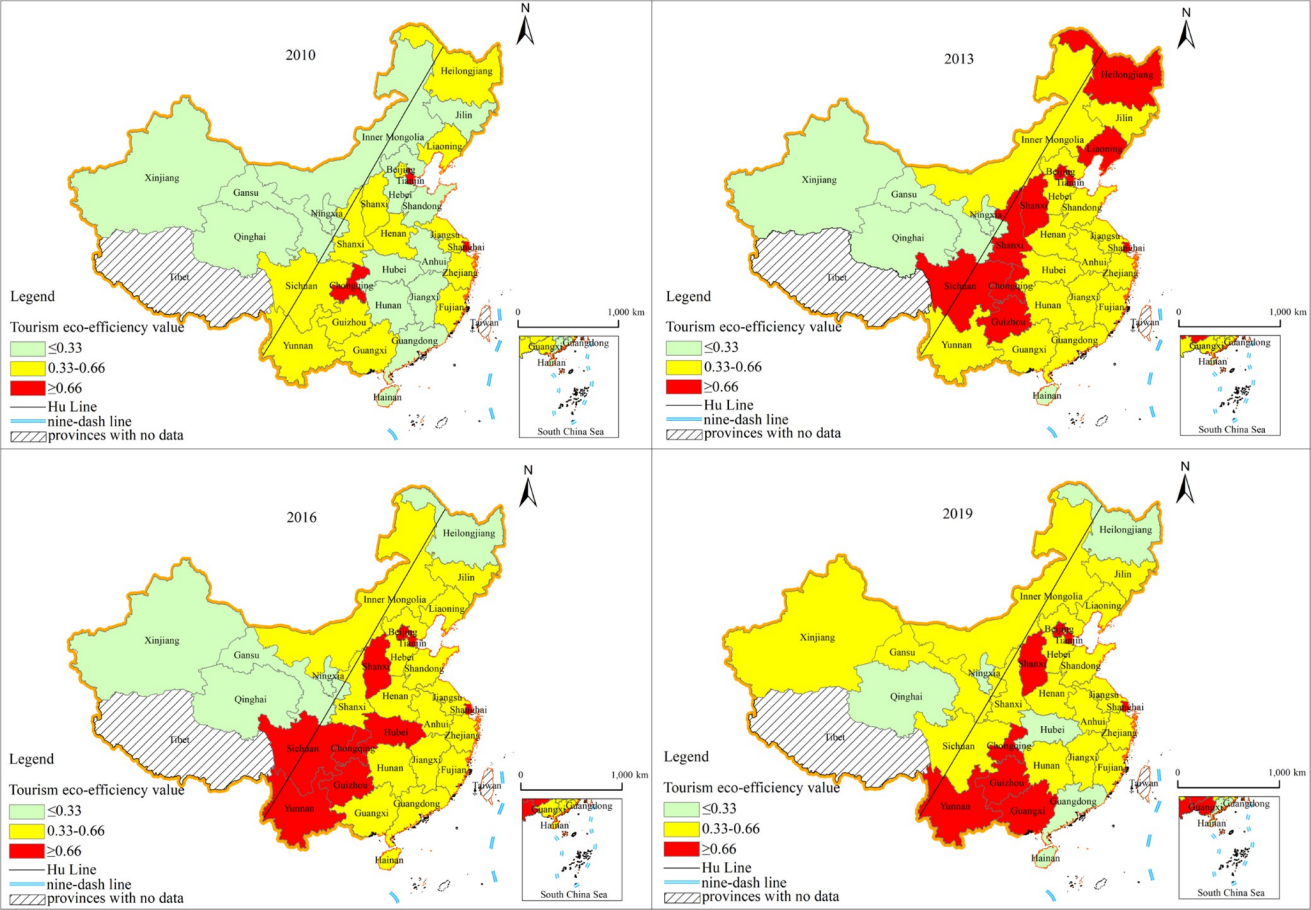
Values of tourism eco-efficiency in China provinces in 2010, 2013, 2016 and 2019 years. (This map is based on the standard map with review number GS(2019)1697 downloaded from the Ministry of Natural Resources Standard Map Service website (http://bzdt.ch.mnr.gov.cn), and the base map has not been modified).

As shown in Fig 3, we refer to the efficiency classification criteria of existing studies and use 0.33 and 0.66 as the threshold values to classify tourism eco-efficiency into three levels: low, medium and high efficiency [55]. The high-efficiency regions, with the cities of Chongqing, Beijing, and Tianjin and the provinces of Guizhou and Shanxi as the core, are gradually shrinking. Low-efficiency regions, represented by Qinghai Province, Ningxia Hui Autonomous Region and Hainan Province, are also shrinking. However, the tourism eco-efficiency differential among China’s provinces still exists.

Generally, China’s tourism eco-efficiency values have increased overall. During 2010–2013, high-efficiency provinces increased rapidly and spread from southwest to northeast along the Hu line; while medium-efficiency provinces showed a slow growth trend and gradually covered the area east of the Hu line; low-efficiency provinces declined rapidly, shifting from residing on both sides of the Hu line in 2010 to the left side in 2013. During 2013–2016, the pattern of tourism eco-efficiency remained. From 2016 to 2019, the number of high, medium and low efficiency provinces remained stable, but the spatial pattern changed, tourism eco-efficiency broke through the shackles of Hu line, and the situation of "high in the east and low in the west" was eased.

### 3.2 Evolution of China’s provincial tourism eco-efficiency network structure

**Fig 4:**
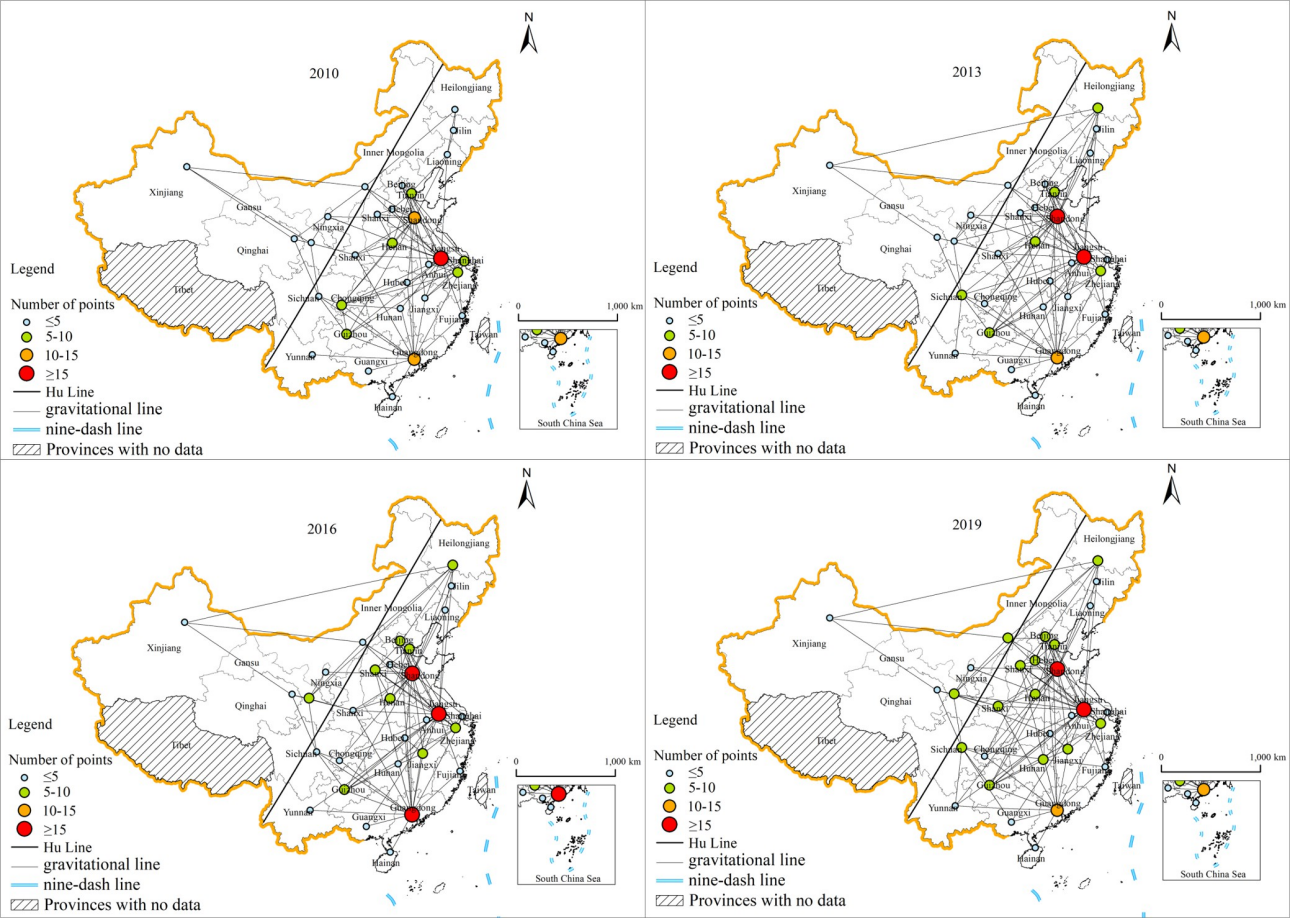
Spatial correlation network of provincial tourism ecological efficiency of China in 2010, 2013, 2016 and 2019. (This map is based on the standard map with review number GS(2019)1697 downloaded from the Ministry of Natural Resources Standard Map Service website (http://bzdt.ch.mnr.gov.cn), and the base map has not been modified).

In this research, ArcGIS 10.8 software was used to create a spatial network correlation map of China’s provincial tourism eco-efficiency for 2010, 2013, 2016 and 2019 based on the gravitational binary matrix. As shown in Fig 4, during the research period, the spatial network of provincial tourism eco-efficiency in China showed multithreaded, dense, and diversified characteristics. Moreover, due to their large volume of tourism and ecological economic development, the eastern provinces of China—Guangdong, Shandong, and Jiangsu—have long been important nodes driving the growth of tourism in neighboring provinces. With the rapid development of the tourism economy, the scale of tourism eco-cooperation among provinces is increasing, and the spatial network of provincial tourism eco-efficiency is becoming increasingly complex.

#### 3.2.1 Overall network characteristics analysis

1. Spatial linkage intensity: network density. As shown in Fig 5 (left), the trends of network density and correlations of provincial tourism eco-efficiency in China remained consistent from 2010 to 2019, essentially showing an N-shaped trend, that is, “increasing, then decreasing and then increasing”. Overall, network density increased from 0.155 in 2010 to 0.174 in 2019, and the number of network correlations increased from 135 in 2010 to 151 in 2019. This result indicates that the overall spatial correlations among provinces have been further strengthened, but the degree of correlation remains low. This relationship might be caused by the national tourism policy, which more closely links China’s tourism eco-efficiency among provinces. However, the new period of tourism industry development focuses on both speed and quality, and there is a downward trend as provinces and municipalities focus more on internal tourism collaboration and reorganization when restructuring their tourism industry.

**Fig 5:**
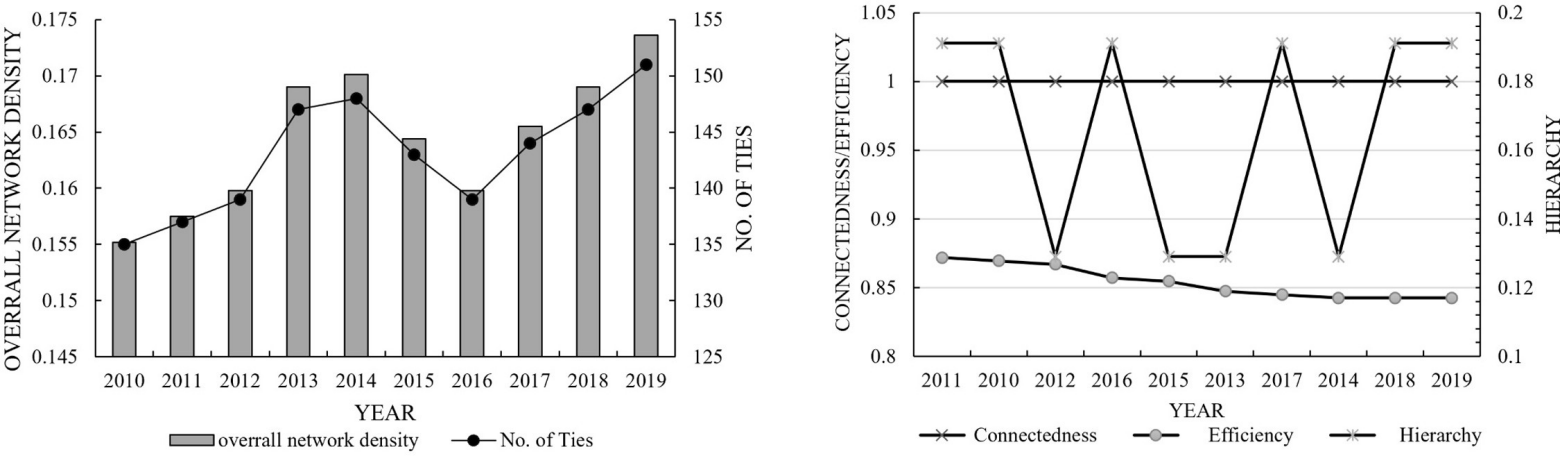
The overall network characteristics of China’s provincial tourism eco-efficiency.
2. Spatial network relevance: connectedness, hierarchy and efficiency. As shown in Fig 5 (right), network connectedness remained at 1 during the research period, indicating that the overall network structure has good connectivity, accessibility and robustness. The network hierarchy was on the low side, fluctuating between 0.129–0.191. This result indicates that there is still a “hierarchical” spatial network structure in China’s provincial tourism eco-efficiency. Network efficiency decreased from 0.871 in 2010 to 0.842 in 2019, which indicates multiple overlapping spillover channels among network nodes, continuous strengthening of interprovincial spatial association, and gradual improvement of network stability.

#### 3.2.2 Individual network characteristics analysis

1. Core node roles: degree centrality. This result indicates the number of points connected to a province in the overall network and characterizes its "rights". The indegree variable indicates the aggregation and polarization effects of provinces on other elements in the overall network. The outdegree indicates the radiation and spillover effects of provinces on elements in other provinces in the overall network. During 2010–2019, the degree of centrality of the provinces changed considerably, and the regional tourism eco-efficiency strongly broke the “east high and west low” nonequilibrium pattern. In 2010, Chongqing city in western China and Guangdong Province in eastern China played important roles in China’s provincial tourism eco-efficiency spatial network, while in 2019, the eastern provinces of Jiangsu, Shandong and Guangdong were far ahead in terms of degree centrality. In terms of indegree and outdegree, Jiangsu, Shandong and Guangdong provinces were in the leading positions in 2010 and 2019. Their location in China’s coastal region offers obvious advantages in tourism resources, comprehensive tourism transportation facilities and high circulation of factors. A better tourism economy has a siphoning effect in these regions and will continue to be an important part of the country’s spatial network of provincial tourism eco-efficiency. Correspondingly, the outdegrees of all provinces were greater than 0 in 2010 and 2019, indicating a spatial radiation effect of tourism eco-efficiency in each province.
2. Network node function: closeness centrality. It characterizes the "distance" between a province and other provinces in the network. As shown in Table 3, the closeness centrality in 2010 and 2019 was mainly concentrated in [38, 39]. Their distribution was relatively balanced. In both 2010 and 2019, Jiangsu Province had the highest level of closeness centrality, standing out as the core of the overall network. Diversified tourism products and multiple tourism lines have vertically deepened the breadth and depth of tourism efficiency radiation, indicating that Jiangsu Province tourism resources are endowed with high tourism economic benefits. The interaction and complementarity of tourism development, coupled with its superior location advantage, makes this province an important "bridge" for interregional tourism exchanges and cooperation.

**Table 3:** Centrality characteristics of spatial correlation network of tourism eco-efficiency of China in 2010 and 2019.

Province	2010					2019				
	InDegree	OutDegree	Degree	Closeness	Betweenness	InDegree	OutDegree	Degree	Closeness	Betweenness
Beijing	5	5	20.690	54.717	2.446	8	6	34.483	59.184	2.353
Tianjin	7	4	10.345	54.717	1.68	7	4	24.138	53.704	0.533
Hebei	4	4	13.793	50	0.137	5	4	17.241	50.877	0.131
Shanghai	4	3	24.138	51.786	0.755	4	3	13.793	50.877	0.393
Jiangsu	17	17	20.690	69.048	23.703	22	20	75.862	78.378	41.352
Zhejiang	4	7	13.793	49.153	0.86	4	8	27.586	53.704	0.883
Fujian	2	2	6.897	44.615	0.053	4	2	13.793	50.877	0.911
Shandong	17	16	24.138	65.909	26.789	13	16	58.621	65.909	13.865
Guangdong	10	16	55.172	61.702	20.476	8	14	48.276	60.417	11.916
Hainan	1	1	3.448	38.667	0	1	1	3.448	38.158	0
Shanxi	7	5	17.241	54.717	1.68	7	5	24.138	53.704	0.923
Anhui	4	4	13.793	51.786	0.755	4	3	13.793	50.877	0.393
Jiangxi	6	5	10.345	53.704	1.535	6	6	20.69	52.727	2.41
Henan	4	6	20.690	51.786	0.421	3	6	20.69	53.704	0.277
Hubei	4	4	13.793	51.786	0.461	2	4	13.793	51.786	0.475
Hunan	2	4	13.793	48.333	0.103	3	5	17.241	52.727	0.45
Inner mongolia	2	4	6.897	50.877	3.571	3	5	20.69	54.717	3.602
Guangxi	2	1	6.897	44.615	0.053	3	2	10.345	50	0.794
Chongqing	4	4	65.517	52.727	0.718	4	4	17.241	53.704	2.128
Sichuan	7	4	24.138	54.717	6.003	4	7	24.138	48.333	2.229
Guizhou	7	4	24.138	55.769	2.529	8	3	27.586	56.863	2.885
Yunnan	1	2	24.138	42.647	0	4	3	13.793	52.727	1.72
Shaanxi	4	4	13.793	53.704	1.063	4	4	20.69	55.769	3.029
Gansu	5	4	27.586	56.863	6.778	6	3	27.586	56.863	4.58
Qinghai	3	1	11.069	40.845	1.034	4	1	13.793	43.939	0
Ningxia	0	2	10.345	44.615	0	0	3	10.345	49.153	0
Xinjiang	0	3	6.897	38.667	0.452	0	3	10.345	39.189	0
Liaoning	2	3	17.241	42.029	0	2	3	10.345	48.333	0.271
Heilongjiang	6	1	24.138	50.877	4.071	6	1	24.138	52.727	0.855
Ji lin	2	3	10.345	42.029	0	2	3	10.345	48.333	0.271
Mean value	4.767	4.767	18.563	50.780	3.604	5.033	5.067	22.299	52.942	3.321
3. Bridging node function: Betweenness centrality. Most provinces showed a relatively balanced distribution of betweenness centrality, with some small differences. Nevertheless, a few provinces have experienced extreme situations. In 2010 and 2019, the betweenness centrality in the eastern provinces of Jiangsu, Shandong and Guangdong was significantly higher than that in other provinces, and the sum of these three provinces’ betweenness centrality accounted for 70.968% and 67.133% of China’s betweenness centrality, respectively. These three provinces play a powerful role as a bridge and intermediary in the process of the flow and transmission of tourism ecological elements and tourism resources, and the other provinces rely heavily on them. In 2010 and 2019, betweenness remained at 0 for Hainan Province, indicating that there is less communication between it and the other provinces and poor factor circulation.

#### 3.2.3 Core and periphery structure evolution analysis

**Fig 6:**
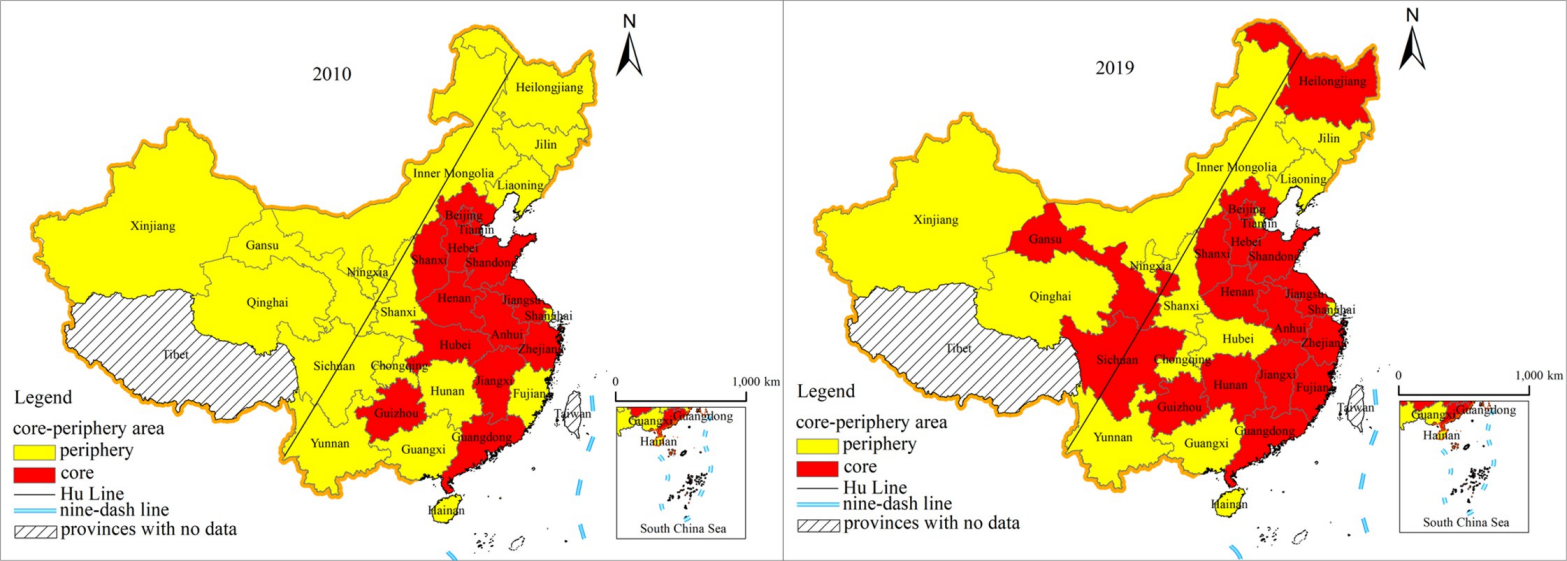
The “core-periphery” structure of China’s provincial tourism eco-efficiency in 2010 and 2019. (This map is based on the standard map with review number GS(2019)1697 downloaded from the Ministry of Natural Resources Standard Map Service website (http://bzdt.ch.mnr.gov.cn), and the base map has not been modified).

The density matrix showed an overall convergence between the network density matrix within the core and periphery areas, with the network density within the core area decreasing from 0.442 to 0.367 and the network density within the periphery area decreasing from 0.063 to 0.033 (Table 4). The network density matrix of the core-to-periphery area and periphery area-to-core area generally showed a dispersion trend. The network density of the core-to-periphery area rose from 0.109 to 0.223, while the network density of the periphery-to-core area rose from 0.131 to 0.223. Furthermore, the core-periphery distribution and evolutionary trends showed that a clustering distribution of core-periphery areas of China’s provincial tourism eco-efficiency. As shown in Fig 6, in 2010, the core area was mainly located east of the Hu Line and included 13 provincial-level administrative units in Beijing city, Tianjin city, Hebei Province, Zhejiang Province, Guangdong Province, Jiangsu Province and Shandong Province in eastern China; Shanxi Province, Anhui Province, Jiangxi Province, Henan Province and Hubei Province provinces in central China; and Guizhou Province in western China. In 2019, the core area was displaced to some extent, and aggregation weakened. Based on the original data, Hubei Province and Tianjin City withdrew from the core camp, while Fujian Province, Gansu Province, Heilongjiang Province, Hunan Province and Sichuan Province joined it. In terms of the number of provincial administrative units occupied by the core fringe, in 2010, there were 13 provincial and urban areas in the core, while in 2019, this figure rose to 16, and overall spatial connectivity increased.

**Table 4:** The core-periphery density matrix of China’s provincial tourism eco-efficiency in 2010 and 2019.

Network Density	2010		2019	
	Core	Periphery	Core	Periphery
Core	0.442	0.109	0.367	0.223
Periphery	0.131	0.063	0.223	0.033

### 3.3 Tourism eco-efficiency network structure driving factors

#### 3.3.1 Factors influencing the provincial tourism eco-efficiency network structure

The sustained optimization of the spatial pattern of tourism eco-efficiency is a combination of multiple factors. The level of economic development directly affects interprovincial cooperation in tourism project investment and tourism infrastructure cobuilding concerning the growth of the tourism eco-efficient network structure, using GDP per capita to represent the regional economic development level [56]. The tourism industry structure objectively reflects the attention of local governments to tourism, and a reasonable tourism industry structure is the key to the ecological tourism economy [57]. The research selected the ratio of total regional tourism revenue to regional GDP to characterize the tourism industry structure [57]. Tourism resources drive the flow of tourism factors, affecting the flow and allocation of tourism input factors between provinces; thus, the choice of different levels of scenic spots was based on weighted representation. The level of information development is the bridge between tourism economic spatial agglomeration and radiation, expressed in terms of total postal and telecommunications services [38, 58]. The application of regional technology innovation and progress in the tourism industry improves the utilization efficiency of tourism energy resources and strengthens the energy-saving and emission-reduction abilities of tourism enterprises [38]. The flow and disposition of technology and personnel promotes regional tourism ecological efficiency, and the energy consumption per unit of tourism income is used to express the level of tourism technology [56].

In this study, we chose 2019 as a representative year in the study period, the intensity of the spatial association of tourism eco-efficiency of provinces as the explanatory variable, and the matrix of economic development level (EDL), tourism industry structure (TIS), tourism resource endowment (TRE), information development level (IDL), and tourism technology level (TTL) as explanatory variables. To avoid the interference of magnitude differences on the data fit, individual data were normalized before the formal data analysis.

#### 3.3.2 Results of QAP correlation analysis and regression analysis of provincial tourism eco-efficiency network structure

The constructed model was used to perform QAP regression analysis on the spatial network of tourism eco-efficiency and the matrix of influencing variables in China’s provincial areas, with the number of permutations set to 2000. As shown in Table 5, the results showed that the adjusted R^2^ = 0.229 and passed the 1% significance level test, indicating that the variation in the selected indicators could explain 22.9% of the spatial correlation of tourism eco-efficiency in China’s provinces. Previous research has shown that QAP models based on the same data generally had lower coefficients of certainty than ordinary least square(OLS) models, and the values of R^2^ in studies based on QAP methods ranged mostly between 12.5% to 40.3%. The R^2^ data of the research were moderate, and the fit of the indicators was good, so it has good explanatory power [59, 60].

**Table 5:** QAP correlation analysis and regression analysis results.

Explanatory variables	QAP correlation analysis		QAP regression analysis	
	Correlation coefficient	*P* value	Regression coefficient	*P* value
Intercept			0.073	0.000***
*EDL*	0.430	0.000***	0.186	0.007**
*TIS*	0.085	0.040*	-0.117	0.054
*TRE*	0.135	0.006**	-0.041	0.241
*IDL*	0.452	0.000***	0.268	0.001***
*TTL*	0.321	0.000***	0.104	0.031*
*R* ^2^			0.229	0.000***
Adj-*R*^2^			0.226	0.000***

Note: *** means *p* < 0.001,

** means *p* < 0.01,

* means *p* < 0.05

The results of the QAP regression analysis were essentially consistent with the correlation analysis, but some of the variables failed the significance test, which is also similar to existing studies [50]. Among all variables that passed the significance level, EDL, IDL and TTL proved to be significant contributors to the development of the spatial network structure of tourism eco-efficiency in China’s provinces. The provincial areas with higher levels of economic development are both destinations and sources of tourists. Tourism flows connect the two regions, allowing tourism components to flow into each other. This flow, coupled with the investment and construction of tourism infrastructure facilities, leads to the spillover of economic resources, thereby strengthening the link between the two regions. The tourism technology level is directly related to its provincial tourism eco-efficient spatial structure development. Technology determines the ecological civilization era of the track; enhances tourists’ sense of participation, experience and access; reduces the pressure of tourism on the environment; and enhances communication and exchange between tourist places. In the era of intelligent tourism, the tourism information level is conducive to the diversion of different tourist destinations, promoting the spatial replacement of passenger flows among provincial and urban areas and thus improving the level of tourism efficiency and spatial network correlation.

It should be noted that TIS and TRE did not pass the 5% significance test. The tourism industry structure laterally reflects the importance that local governments attach to the tourism industry, whereas the disparate tourism industry status does not facilitate cooperation between developed and backward tourism regions, preventing the development of a spatial network structure of tourism eco-efficiency in China’s provinces. Tourism resources, as the native attraction of tourists, largely determine the level of development of tourist destinations, yet the lack of scientific planning and long-term calculation of tourism resources has led to a low level of attractiveness for tourism resources. Furthermore, tourism resource development project planning ideas and planning principles are common, and development ideas and concepts are similar, resulting in excessive homogenization of tourism, which is not conducive to interregional tourism cooperation and exchange.

## 4 Conclusion

The study combines the undesired output Super-SBM model and SNA to determine the eco-efficiency of provincial tourism in China from 2010–2019 and analyze its spatial correlation characteristics and its influencing factors. The main findings indicate the following:

1. The mean value of provincial tourism eco-efficiency in China has been maintained at 0.405~0.612, with an overall fluctuating upward trend. The tourism eco-efficiency of eastern China is higher than that of the central, western and northeastern regions, which have not formed a stable spatial distribution pattern.
2. The spatial network of provincial tourism eco-efficiency in China is multithreaded, dense and diversified. Throughout the whole network, network affiliations are becoming closer, and network structure robustness, which still has a "hierarchical" spatial network structure, is gradually improving. In individual networks, Jiangsu, Guangdong and Shandong provinces in eastern China have a higher degree of centrality, closeness centrality and betweenness centrality than other provinces, indicating their dominance within the network. Hainan Province, also located in eastern China, has not yet built a "bridge" for tourism factor circulation. In the core-periphery model, the core-periphery areas of China’s provincial tourism eco-efficiency are distributed in clusters, and the number of "core members" has increased.
3. The economic development level, information technology development level, and tourism technology level collectively drive the development and evolution of China’s provincial tourism eco-efficiency spatial network.

## 5 Discussion

### 5.1 Policy recommendations

Based on the above findings, this study makes the following recommendations. First, a sound mechanism for regional tourism cooperation should be established. Provinces should overcome the restrictions of administrative divisions and foster the mutual promotion of tourism routes, mutual delivery of tourism sources, sharing of tourism resources, and interoperability of tourism information in eastern, central, western and northeastern China to help improve the country’s overall tourism eco-efficiency. Second, a tourism information interaction mechanism should be established. Building up tourism information interaction, integrating various types of tourism information among the different tourism regions, linking the performance of regional tourism resource diversity and complementary features, and converting tourism into regional tourism flows would promote the common development of tourism destinations in each tourism region. Third, it is recommended that internet technology be adopted to promote the upgrading of regional tourism technology. To enable tourism enterprises to develop new products, services and business models, relying on technologies such as virtual reality (VR), augmented reality (AR) and 5G combined with different regional tourism culture IPs would create a combination of virtual and real "cloud tourism", "cloud live", and "cloud exhibition", achieving a broader range of regional tourism resource cooperation.

### 5.2 Limitations and future research

This research constructed a binary matrix based on the modified gravity model, and the calculation process primarily considered spatial distance. However, with the diversity and sophistication of transportation modes, tourists’ perception of spatial distance has weakened while their perception of temporal distance has increasingly strengthened, so temporal distance urgently needs to be incorporated into the modified gravity model. In addition, QAP regression analysis and correlation analysis were used to investigate the influencing factors of the spatial network of eco-efficiency of China’s provincial tourism. The five major variables of EDL, TIS, TRE, IDL, and TTL were analyzed, but the impact of potential influencing factors on the development of the spatial network has yet to be explored. Furthermore, the linkage effect among variables exerts an impact on the results and is also in urgent need of verification.

## Supporting information

S1 Table Tourism eco-efficiency. (XLSX)
